# Case Report: Fanconi-Bickel Syndrome in a Chinese Girl With Diabetes and Severe Hypokalemia

**DOI:** 10.3389/fped.2022.897636

**Published:** 2022-06-09

**Authors:** Hongbo Chen, Juan-juan Lyu, Zhuo Huang, Xiao-mei Sun, Ying Liu, Chuan-jie Yuan, Li Ye, Dan Yu, Jin Wu

**Affiliations:** ^1^Department of Pediatrics, West China Second University Hospital, Sichuan University, Chengdu, China; ^2^Key Laboratory of Birth Defects and Related Diseases of Women and Children, Ministry of Education, Sichuan University, Chengdu, China

**Keywords:** case report, *SLC2A2*, GLUT2, Fanconi-Bickel syndrome, hypokalemia, diabetes

## Abstract

Fanconi-Bickel syndrome (FBS) is a rare autosomal recessive carbohydrate metabolism disorder. The main symptoms of FBS are hepatomegaly, nephropathy, postprandial hyperglycemia, fasting hypoglycemia, and growth retardation. Hypokalemia is a rare clinical feature in patients with FBS. In this study, we present a neonate suffering from FBS. She presented with hypokalemia, dysglycaemia, glycosuria, hepatomegaly, abnormality of liver function, and brain MRI. Trio whole-exome sequencing (WES) and Sanger sequencing were performed to identify the causal gene variants. A compound heterozygous mutation (NM_000340.2; p. Trp420*) of *SLC2A2* was identified. Here, we report a patient with FBS in a consanguineous family with diabetes, severe hypokalemia, and other typical FBS symptoms. Patients with common clinical features may be difficult to diagnose just by phenotypes in the early stage of life, but WES could be an important tool. We also discuss the use of insulin in patients with FBS and highlight the importance of a continuous glucose monitoring system (CGMS), not only in diagnosis but also to avoid hypoglycemic events.

## Introduction

Fanconi–Bickel syndrome (FBS, OMIM#227810) is a rare autosomal recessive disease characterized by impaired liver glucose homeostasis and proximal renal tubular dysfunction (1). Fanconi and Bickel first reported this disease in 1949 (2). FBS characteristically involves Fanconi syndrome with glycosuria, galactosuria, aminoaciduria, proteinuria, and phosphaturia. Other characteristics include shortness, rickets, poor growth, hepatomegaly, glucose, galactose intolerance, etc. (3) However, not all patients will develop typical clinical signs, including isolated glucosuria and hypokalemia (4, 5). A high index of suspicion is needed for diagnosis due to its varied clinical presentations. It is mainly based on the clinical symptoms, radiological and biochemical features of rickets, laboratory data on renal tubular dysfunction, resultant metabolic acidosis, accumulation of glycogen on the liver or renal biopsy (6), and whole-exome sequencing (WES).

The solute carrier family 2 member 2 (*SLC2A2*) gene is associated with FBS. *SLC2A2* codes for the glucose transporter protein 2 (GLUT2) expressed in the liver, intestine, pancreas, and kidney (7), which contains 11 exons and plays an important role in the characteristic affinity for glucose (8–10).

Up to date, there are more than 100 patients and 80 *SLC2A2* variants that have been reported to cause FBS across the globe; less than ten patients with FBS have been reported in China. In our study, we present a Chinese patient affected with FBS who had diabetes and severe hypokalemia. This patient had a hereditary mutation of *SLC2A2*, and she presented with poor growth, hepatomegaly, short stature, rickets, glycosuria, proteinuria, and phosphaturia. We also discuss the use of insulin, diabetes, severe hypokalemia, and the importance of a continuous glucose monitoring system (CGMS) in diagnosis.

## Narrative

The propositus was born at 41 weeks as the first child of consanguineous parents. She had no family history. Her birth weight was 2,780 g with no evidence of intrauterine growth retardation. She was immediately breastfed, and supplementary food was added at 4 months of age. Her history was notable for hospitalization for neonatal pneumonia and neonatal hyperbilirubinemia at age 5 days. She had received the first dose of hepatitis B vaccine, BCG vaccine, and polio vaccine. She was able to look up at 4 months and was turning over (tummy to back only) at 6 months. At her initial examination, she could not sit stably.

At the age of 4 months, her urine became thick and foamy without polydipsia, polyuria, fever, vomiting, or diarrhea. She was sent to a local hospital and her routine urine test showed urine glucose 4+, urine protein 2+, ketone body 1+, and urine specific gravity 1.05. Her alanine aminotransferase (ALT) level was 66 U/L, and her aspartate aminotransferase (AST) level was 196 U/L. Routine blood and glucose tests were within normal limits (WNL). She did not receive any medication or other therapy for 2 months.

**FIGURE 1:**
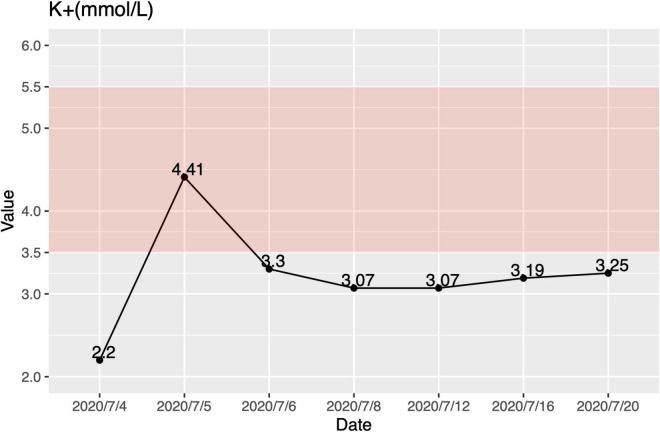
Hypokalemia in this patient. The lowest serum potassium was 2.2 mmol/L. Oral potassium supplementation (100 g potassium citrate mixed with 1,000 ml water, 5 ml PO. Tid.) could not keep her blood potassium at normal levels. The pink area is the normal range.

At the age of 6 months, her urine characteristics remained thick and foamy. Additionally, she got a worse liver function and hypokalemia (ALT 508 U/L, AST 498 U/L, K + 2.95 mol/L). She was suggested to our hospital for further treatment immediately. In our patient, the lowest serum potassium was 2.2 mmol/L at the first sight, accompanied by hypocalcemia and hyperchloremia (Figure 1). She was treated with intravenous and oral potassium supplementation immediately, and the value of potassium levels increased to 4.41 mmol/L 12 h later. However, oral potassium supplementation (100 g potassium citrate mixed with 1,000 ml water, 5 ml PO. Tid) could not keep her blood potassium at normal levels. At the first physical exam, she had a bodyweight of 6 kg (Z-score = 2.03, 2.1%tile), length of 68 cm (Z-score = 0.48, 68.4%tile), and a body mass index of 12.98 (Z-score = 2.96, 0.1%tile). Her subcutaneous fat was thin, and there were no abnormalities of the skin and mucosa. Her abdomen was soft, with a maximum abdominal circumference of 38 cm. The liver was soft and 6 cm below the ribs, and the spleen was 2 cm below the ribs. Examinations of the nervous and cardiovascular systems were within normal limits (WNL). There were no other significant pathological signs.

**FIGURE 2:**
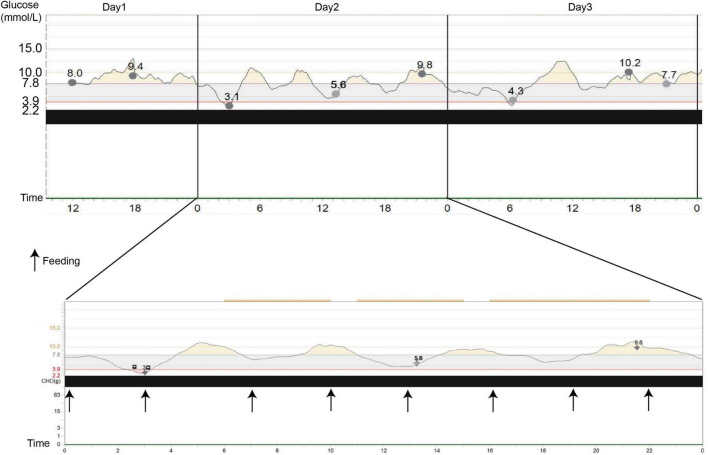
Continuous glucose monitoring system (CGMS) at diagnosis. The patient presented postprandial hyperglycemia, and when she fasted, a tendency to hypoglycemia, followed by marked hyperglycemia, can be observed. Black arrows represent the meals.

In our hospital, her laboratory findings also revealed elevated levels of hepatic transaminases (ATS/ALT), proteinuria, and glucosuria (Table 1). Urinary microproteins (α1 microglobulin, microalbumin) and electrolytes were significantly increased, but the urea and creatinine were WNL, revealing proximal renal tubular dysfunction without proximal tubular acidosis. Urine output was 15 cc/kg/h in 10 h. The 24-h urinary protein excretion was not completed due to the difficulty in sampling. The patient’s parents refused a liver biopsy. Fingertip blood glucose was higher than 11.1 mmol/L several times, we did continuous glucose monitoring and found preprandial hypoglycemia and postprandial hyperglycemia (Figure 2). Other symptoms, which cannot be explained by neonatal diabetes (such as hypoglycemia without insulin, obvious enlargement of liver and spleen, electrolyte disturbance, normal range of HbA1c, etc.), and other metabolic diseases, were considered. Parathyroid hormone, glycosylated hemoglobin, the complete set of transfusion immune functions, all forms of hepatitis, renal function, autoantibody, Epstein-Barr virus nucleic acid, Epstein-Barr virus antibody spectrum, TORCH (a panel which consists of antibodies to toxoplasmosis, rubella, cytomegalovirus, and herpes simplex virus), and stool examination were all WNL. No abnormality was found in hematuria tandem mass spectrometry.

**TABLE 1:** Laboratory findings of the patient with Fanconi-Bickel syndrome (FBS).

Type	Value	Unit
**2020-07-04**		
25(OH)D (25-hydroxyvitamin D)	14.8	ng/mL
Albumin	36.3	g/L
Bilirubin, conjugated (direct)	0.1	μmol/L
Bilirubin, total	6.4	μmol/L
BUN (blood urea nitrogen)	0.71	mmol/L
Calcium	1.95	mmol/L
Chloride	112.5	mmol/L
Creatinine	13	μmol/L
Gamma-glutamlytransferase (GGT)	1079	U/L
Glucose	4.6	mmol/L
Hemoglobin A1C (HbA1C)	5.3	%
Magnesium	0.89	mmol/L
Phosphorus, serum	0.48	mmol/L
Potassium	2.2	mmol/L
Protein, total	62.4	g/L
parathyroid hormone (PTH)	39.3	pg/mL
Sodium	136	mmol/L
**2020-07-05**		
Alanine aminotransferase (ALT)	135	U/L
Aspartate aminotransferase (AST)	582	U/L

**FIGURE 3:**
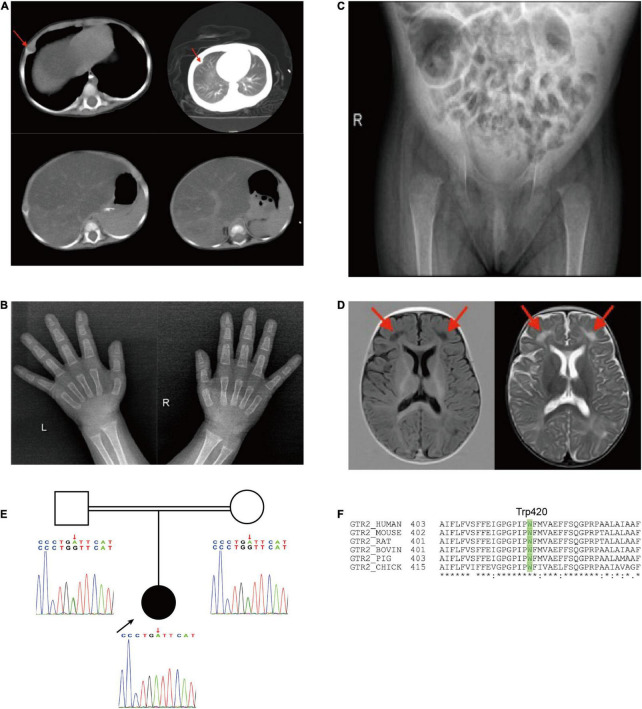
**(A)** Chest CT showing that the cartilaginous end of the rib was enlarged; Abdominal CT showing an uneven decrease of liver density; **(B)** X-ray of both hands and **(C)** X-ray of hip joint indicated rickets; **(D)** brain MRI, low signal on T1 and high signal on T2. **(E)** Pedigree showing SLC2A2 homozygous mutations in the child. **(F)** Trp420 is conserved across species.

Contrast-enhanced computed tomography (CT) confirmed that the cartilaginous end of the rib was enlarged (red arrows), as well as the presence of hepatomegaly and reduced liver density (Figure 3A). There was no evidence of morphological abnormalities in the kidney or other organs. The X-ray of her hands showed that the density is reduced, and the central part is depressed, especially on the left side (Figure 3B). The X-ray of her hip joint showed that the epiphysis of the bilateral femoral head does not appear, and the bone density of the bilateral acetabulum is reduced so that active rickets was considered (Figure 3C). A brain magnetic MRI scan showed multiple symmetrical signal abnormalities in the subcortical white matter of the bilateral cerebral hemisphere (red arrows in Figure 3D). After our radiologist read her MRI images, the level of brain myelination was slightly lower than that of children of the same age, but she did not have nervous symptoms or seizures.

Subsequent genetic sequencing revealed a homozygous c.1260G > A (p. Trp420*) in exon 10 of *SLC2A2* (NM_000340) after her parents signed the consent form for genetic testing, which supported our diagnosis. Both parents were found to be heterozygous for the same mutation (Figure 3E) and Trp420 is highly conserved across species (Figure 3F). We used the American College of Medical Genetics and Genomics guidelines in 2015 (11) to establish that the variant in *SLC2A2* is likely pathogenic and the ACMG criteria is PVS1 + PM2. Sanger sequencing was performed to confirm the findings. The results of a mitochondrial gene test were negative.

## Discussion

In this case report, we describe a case of FBS with abnormal urination and multiple system involvement. In her clinical symptoms, diabetes and hypokalemia are rare. In this patient, transient or permanent neonatal diabetes is an unusual feature of Fanconi–Bickel syndrome, reported in 15 patients with homozygous *SLC2A2* mutations (3, 6, 12–17). Furthermore, *SLC2A2* mutation has been shown to increase the risk of developing type 2 diabetes (T2D) (18, 19). Hypokalemia has previously been reported in one study (4), but we did not know the accurate value of potassium levels in that case. In our patient, the lowest serum potassium was 2.2 mmol/L, accompanied by hypocalcemia, hyperchloremia, and hypophosphatemia. Hypokalemia does not occur in every patient with FBS, and the relationship between FBS and potassium homeostasis is unclear. She took mother’s milk normally without vomiting, the possible mechanism may be that renal tubular dysfunction leads to increased excretion of potassium from urine. Regardless of the causes, a severe electrolyte disorder may be fatal. Therefore, we suggest routine testing of electrolytes when following up on children with FBS. In addition, we first report abnormality in brain MRI of patients with FBS. Karamizadeh et al. (20) reported a patient with FBS with normal brain MRI. Meanwhile, another Japanese patient with *SLC2A2* c.1159G > A, p. Trp420* mutation also had bilateral basal ganglia calcification shown on CT scan (21). The majority of patients with FBS did not conduct brain MRI. According to previous research, GLUT2 is required for glucose sensing by cells of the central nervous system (22), whether it would cause abnormal accumulation in the brain needs further study.

The mechanisms underlying dysglycaemia in patients with FBS are not well-understood. GLUT2 is mainly expressed in tissues playing important role in glucose homeostasis. During fasting, impaired glucose export due to GLUT2 deficiency in hepatocytes causes hypoglycemia. The glycosuria from the renal tubular leak aggravates the hypoglycemia (7, 23). GLUT2 is considered the major transporter of glucose in the human liver. GLUT2 dysfunction in the liver can lead to hyperglycemia due to decreased response and sensitivity of hepatocytes to insulin signaling, resulting in reduced inhibition of glucose production (24). In addition, GLUT2 deficiency in the pancreatic β-cell impairs insulin secretion, leading to hyperglycemia (23).

The FBS is very rare, especially in Asian populations, including the Chinese (23). The clinical features of patients with FBS frequently overlap with those of other conditions, such as mitochondrial disease, glycogen storage disease, and tyrosinemia type I. Clinical features and CGMS are helpful in the differential diagnosis of these diseases. However, mutation analysis of the *SLC2A2* gene may be useful to establish an FBS diagnosis if a clinical diagnosis is uncertain. This article is the first report on the *SLC2A2* gene mutation Trp420* in China, and this mutation has not been presented in the HGMD database.^1^ Patients with FBS in China are all identified in infancy, while late-onset FBS is seen in foreign countries (25). A case was reported in a 21-year-old Japanese male with hepatorenal glycogen storage, hypoglycemia, hypergalactosemia, and proximal renal tubular dysfunction, which involved a different cDNA mutation (c.1159G > A, p. Trp420*) (21). As in our case, his parents were close relatives. His clinical manifestations included hepatomegaly, diabetes, increased liver glycogen, and galactosemia. At the age of 20, he had a CT scan that showed bilateral basal ganglia calcification and an ultrasound that detected renal calcification. In contrast, our patient did not have galactosemia, basal ganglia, and renal calcification because of her young age. However, she did have an abnormal liver function, bone abnormalities, and severe electrolyte disorders, among other clinical features.

Whether to use insulin to control hyperglycemia is still inconclusive. Taha et al. (26) reported that the use of insulin should be avoided in patients with FBS to decrease the risk of hypoglycemia. In contrast, insulin was used in some patients with hyperglycemia or neonatal diabetes, and all of them did not have symptomatic hypoglycemia (6, 17, 20, 27). In our patient, we use a small dosage of insulin to control postprandial blood sugar and CGMS to monitor for 24 h. The CGMS played an important role not only in diagnosis but also in the prevention of hypoglycemia. (28) Therefore, the requirement of the use of insulin could be decided with the follow-up and monitoring of these patients and future studies (29). Besides insulin, other treatments included sodium dihydrogen phosphate and disodium hydrogen phosphate, vitamin D, and potassium citrate.

The growth of children with FBS will likely be seriously affected (30). In addition to abnormal glucose metabolism, patients with FBS may have repeated pulmonary infections and gastroenteritis attacks (6). Our patient’s body length and weight were normal at birth, but her weight was at the 2.1%tile, and her body mass index (BMI) was at the 0.1% tile at the time of treatment. This finding indicated that her growth and development had been impaired after birth.

Nutrition support, preventing hypoglycemia and hypokalemia, and recovery of caloric-related height or length defects are the primary treatment goals of patients with FBS in early life. The severity of the growth disorder in FBS is more significant than that of other diseases with proximal tubular dysfunction. This indicates that impaired liver glucose homeostasis might contribute to poor growth in patients with FBS. However, intensive nutritional intervention, such as nocturnal enteral nutrition, could be used to treat patients with FBS with growth failure. Ultimately, their final growth parameters could be WNL (30). The prognosis of FBS is relatively favorable through symptomatic and supportive treatment with an improved health conditions in both female and male patients (31, 32).

In conclusion, we are reporting a Chinese patient with FBS with diabetes and severe hypokalemia who had a variable clinical presentation and a novel genetic mutation. Urine and blood tests suggesting abnormal glucose metabolism could be the clues for FBS in neonates and infants. Genetic sequencing is indispensable for diagnosis. Since there is a diversity of disease presentation and severity, early identification, and long-term follow-up could improve the life quality of patients and decrease mortality. Management of FBS is largely supportive and focused on the treatment of rickets and acid-base disturbance and severe electrolyte, and long-term follow-up is also needed.

## Data Availability Statement

The datasets for this article are not publicly available due to concerns regarding participant/patient anonymity. Requests to access the datasets should be directed to the corresponding author.

## Ethics Statement

The studies involving human participants were reviewed and approved by Institute of West China Second University Hospital, Sichuan University. Written informed consent to participate in this study was provided by the participants’ legal guardian/next of kin. Written informed consent was obtained from the individual(s), and minor(s)’ legal guardian/next of kin, for the publication of any potentially identifiable images or data included in this article.

## Author Contributions

HC, J-JL, and ZH collected the clinical data, carried out the initial analyses, and drafted the initial manuscript. X-MS, YL, C-JY, and LY followed up the patients and revised the manuscript. DY and JW reviewed and revised the manuscript. All authors contributed to manuscript revision and approved the final version of the manuscript.

## Conflict of Interest

The authors declare that the research was conducted in the absence of any commercial or financial relationships that could be construed as a potential conflict of interest.

## Publisher’s Note

All claims expressed in this article are solely those of the authors and do not necessarily represent those of their affiliated organizations, or those of the publisher, the editors and the reviewers. Any product that may be evaluated in this article, or claim that may be made by its manufacturer, is not guaranteed or endorsed by the publisher.

## Footnotes

1
http://www.hgmd.cf.ac.uk/ac/index.php
