# Supplementary material for: Case Report: Fanconi-Bickel Syndrome in a Chinese Girl With Diabetes and Severe Hypokalemia
Source: Front Pediatr. 2022 Jun 9;10:897636. doi: 10.3389/fped.2022.897636 (PMC9218529; doi:10.3389/fped.2022.897636)
Supplement: Supplementary file 1 [file Data_Sheet_1.pdf]

Supplementary file

SLC2A2-chr3:170716096-170716096, c.1260(exon10)G>A

Sanger sequencing:

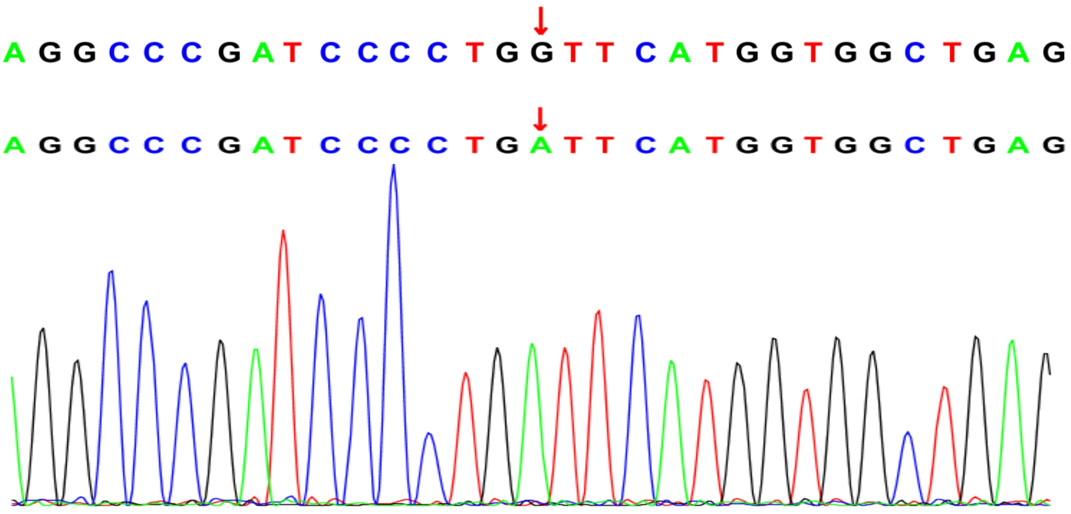

Proband

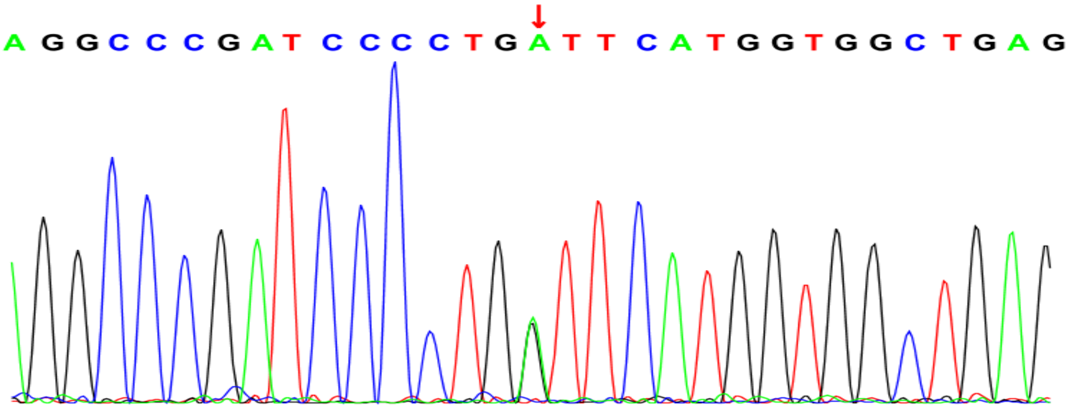

Father

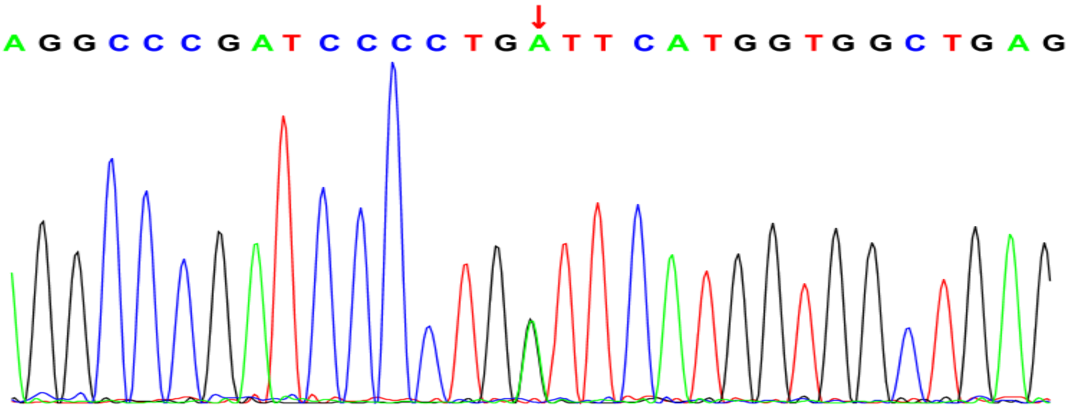

Mother

The BAM file:

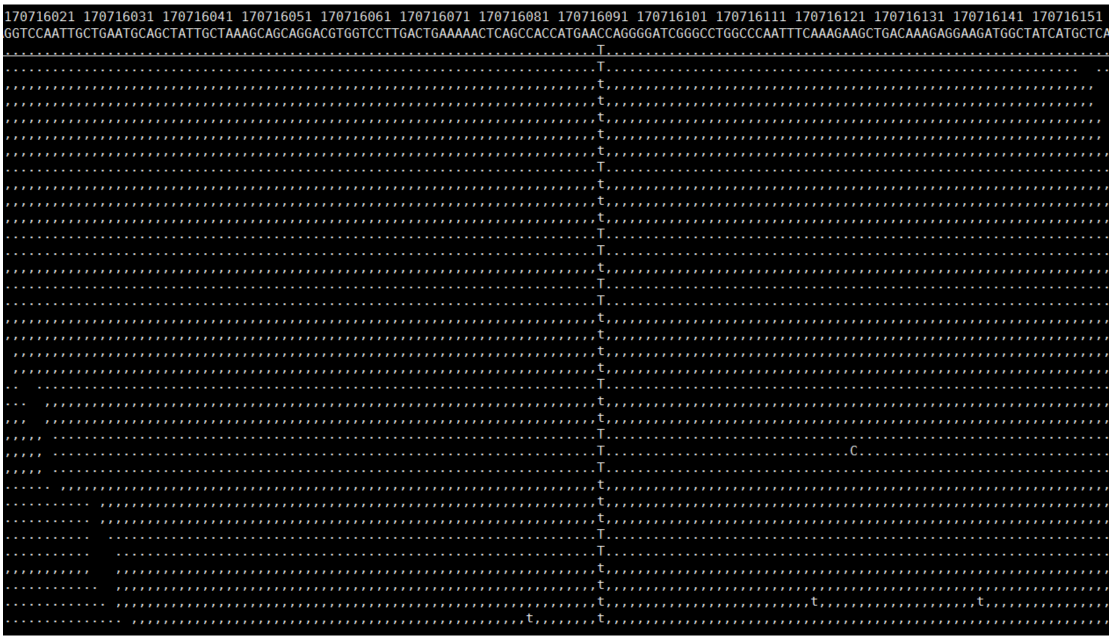

Proband

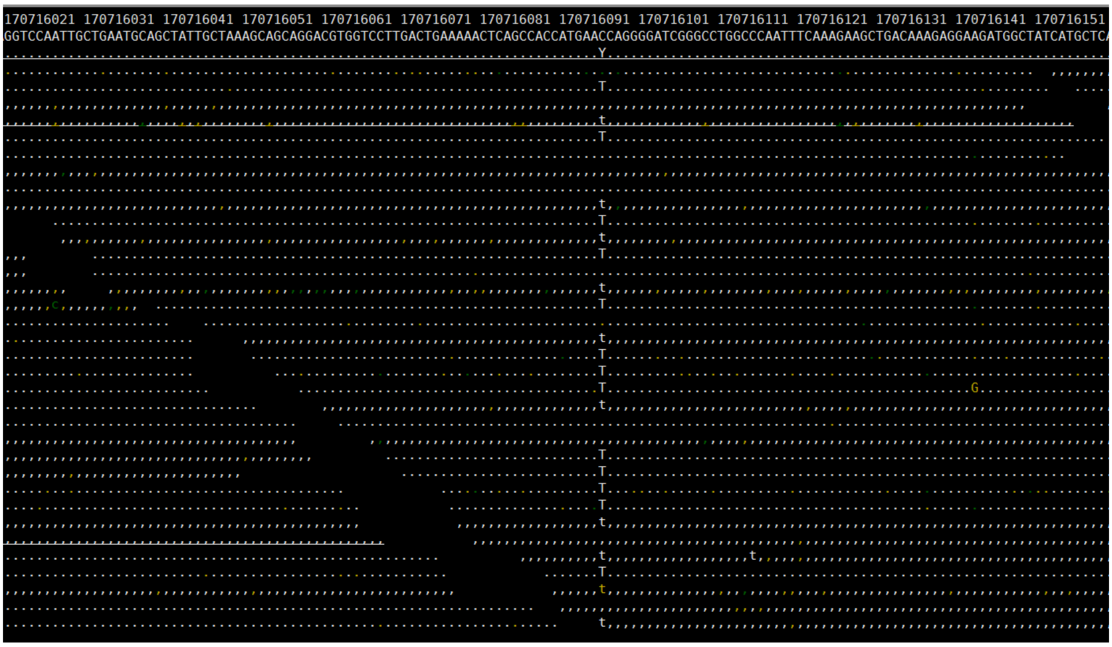

Father

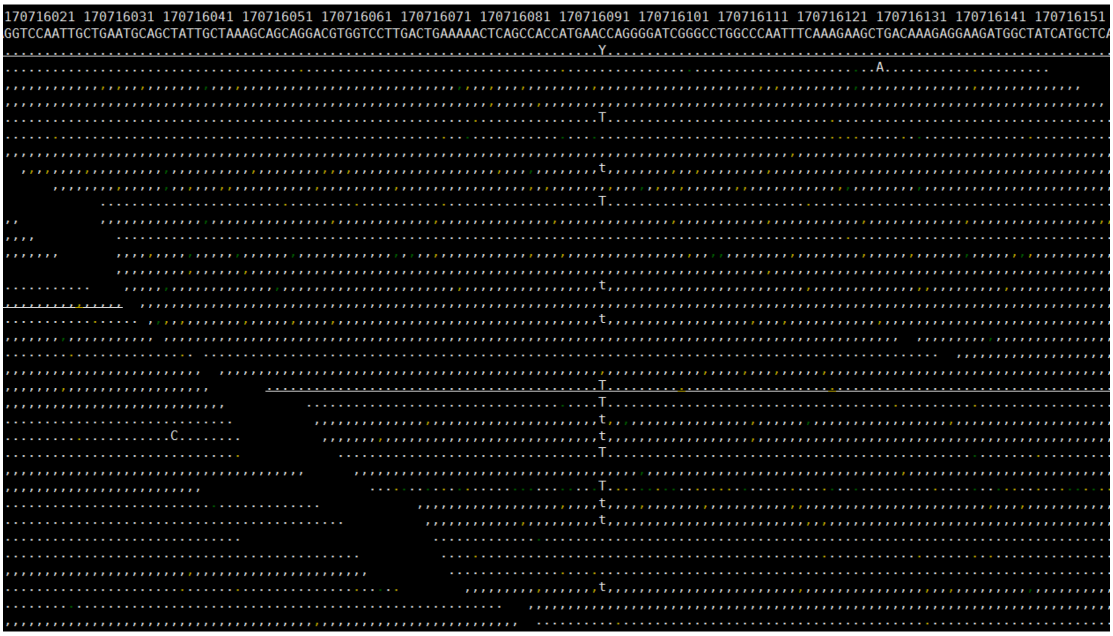
170716021 170716031 170716041 170716051 170716061 170716071 170716081 170716091 170716101 170716111 170716121 170716131 170716141 170716151
GGTCCAATTGCTGAATGCAGCTATTGCTAAAGCAGCAGGACGTGGTCCTTGACTGAAAAACTCAGCCACCATGAACCAGGGGATCGGGCCTGGCCCAATTTCAAAGAAGCTGACAAAGAGGAAGATGGCTATCATGCTCA

Mother
